# Acute phase of Kawasaki disease: a review of national guideline recommendations

**DOI:** 10.1007/s00431-022-04458-z

**Published:** 2022-04-11

**Authors:** Laura Scherler, Nikolaus A. Haas, Anja Tengler, Joseph Pattathu, Guido Mandilaras, André Jakob

**Affiliations:** Department of Pediatric Cardiology and Pediatric Intensive Care, Ludwig-Maximilians-University of Munich, Marchioninistr. 15, 81377 Munich, Germany

**Keywords:** Kawasaki disease, National guideline, Coronary artery aneurysm, COVID-19 MISC-C

## Abstract

Key aspects of the medical management of Kawasaki disease (KD) are not yet supported by a high evidence level, thus making room for individual recommendations. We performed a structured comparison of existing international KD guidelines to analyze potential differences in the implementation of evidence-based KD recommendations regarding diagnosis and therapy. To identify country-specific guidelines, we took a multilateral approach including a comprehensive PubMed literature, online research, and directly contacting national pediatric associations. We then ran a structured guidelines’ analysis and evaluated the diagnostic and therapeutic differences in the context of evidence-based medicine. In this structured guideline analysis, we identified nine national and one European guidelines. According to them all, the diagnosis of KD still relies on its clinical presentation with no reliable biomarker recommended. First-line treatment consistently involves only intravenous immunoglobulin (IVIG) therapy. Recommendations in terms of acetylsalicylic acid, corticosteroids, and additional therapeutic options vary considerably.

*Conclusion*: According to all guidelines, KD is diagnosed clinically with some variance in defining incomplete KD and being a non-responder to treatment. First-line treatment consistently includes IVIG. Recommendations for additional therapeutic strategies are more heterogeneous.

**Table:** 

**What is Known:** *• The diagnosis of KD relies on the clinical presentation, entailing challenges in timely diagnosis.* *• Other treatment options then IVIG are not supported by a high evidence level, making room for individual recommendations.*	
**What is New:** *• Definition of incomplete KD and being non-responsive to an initial treatment vary to some extent between the national guidelines.* *• Only IVIG is consistently proposed throughout all guidelines, further therapeutic recommendations vary between the national recommendations.*

**What is Known:**

*• The diagnosis of KD relies on the clinical presentation, entailing challenges in timely diagnosis.*

*• Other treatment options then IVIG are not supported by a high evidence level, making room for individual recommendations.*

**What is New:**

*• Definition of incomplete KD and being non-responsive to an initial treatment vary to some extent between the national guidelines.*

*• Only IVIG is consistently proposed throughout all guidelines, further therapeutic recommendations vary between the national recommendations.*

## Introduction

Kawasaki disease (KD) is diagnosed worldwide based on the classical clinical symptoms first described by Dr. Tomisaku Kawasaki [1]. If some symptoms are absent, it may be referred to as incomplete KD, with no verifying KD-specific biomarker available. Although inflammation occurs throughout the body, coronary artery involvement can trigger severe coronary artery aneurysms (CAA).

Early treatment options included corticosteroids; however, since they were associated with raising the risk of CAA [2], they have been banned as singular first-line treatment [3]. High-dose acetylsalicylic acid (ASA) is still administered due to its anti-inflammatory effect, but there is no evidence that it prevents CAA development [4]. Only intravenous immunoglobulin therapy (IVIG) is known to significantly reduce CAA rates irrespective of the ethnic background and disease severity [5, 6]. Adding corticosteroids to first-line therapy is again being investigated, but its efficacy in reducing the CAA risk has only been proven in high-risk, IVIG-non-responsive, Japanese KD patients [7]. Further adjunctive therapies for such KD patients have been derived from treatment options for other inflammatory diseases. High-quality trails are rare or still ongoing [8–11].

National disease-specific guidelines should rely on the latest evidence available at the year of publication and in the local setting of the population. The KD evidence however varies not just in the ethnic background. As many aspects are not supported by strong evidence, there is room for individual interpretations. This is currently of special interest, given that young people suffering post-COVID-19 multisystem inflammatory syndrome in children (MIS-C) can present with Kawasaki-like disease, and its treatment is derived from KD treatment options so far [12]. We therefore investigated how national guidelines have adopted the existing evidence to draft their recommendations.

## Method

**Fig. 1:**
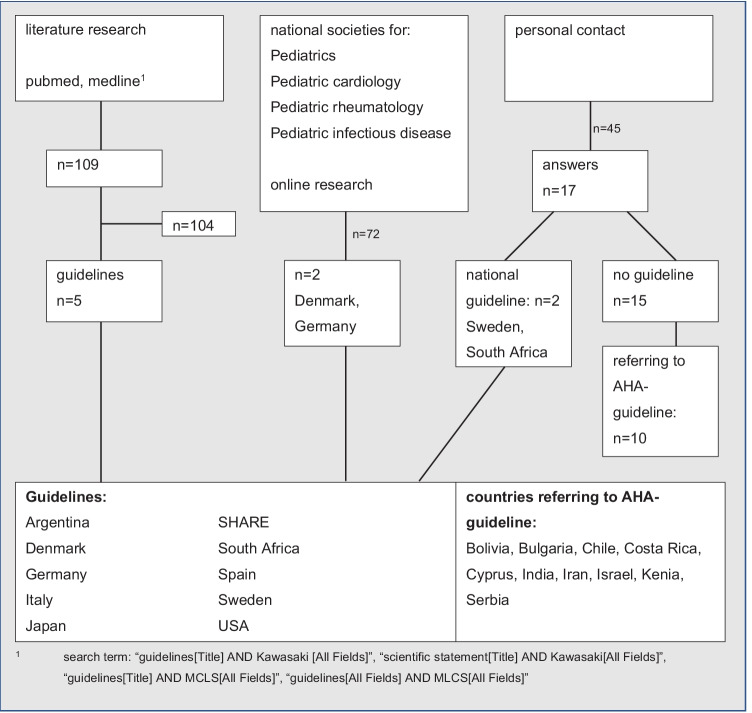
Research. Recruitment flow chart for national guidelines

We took a step-wise approach to identify national guidelines: first, a systematic PubMed and Medline research, including the keywords “Kawasaki disease, Mucocutaneous lymph node syndrome (MCLS), guidelines, scientific statement”; then, a structured search on the official website of national and international pediatric societies including the subspecializations pediatric cardiology, pediatric rheumatology, and pediatric infectious diseases was performed. If those were unsuccessful, we consulted the current directors of each national society concerning the existence of a national KD guideline and, if in case of none, which other country’s guidelines they officially rely on. If we were unable to identify or contact a country-specific society, we directly contacted pediatricians worldwide via a standardized questionnaire (recruitment flow chart; see Fig. 1).

Thus, we identified guidelines from Argentina (AR) [13], Denmark (DK) [14], Germany (DE) [15], Italy (IT) [16, 17], Japan (JP) [18, 19], South Africa (ZA), Spain (ES) [20], Sweden (SE) [21], and the USA (US) [22] and a European guideline from the SHARE initiative [23] (single hub and access point for pediatric rheumatology in Europe) (see Table 1). Guidelines not published in English (AR, DK, ES, DE, and SE) were translated by a professional medical translator. We then conducted a structured analysis to investigate how these issues were addressed in each guideline:

1. Definition and diagnosis of complete, incomplete, and refractory KD
2. First-line treatment
3. Treatment of refractory cases

**Table 1:** National guidelines

Country	Society	Language	Year of publication
Argentina [13] (AR)	Argentine Society of Paediatrics Argentine Society of Cardiology	Spanish	2016
Denmark [14] (DK)	Danish Paediatric Society	Danish	2015
Europe [23]	SHARE initiative^a^	English	2018
Germany [15] (DE)	German Society for Paediatric Rheumatology German Society for Paediatric Cardiology and Congenital Heart Defects	German	2019
Italy [16, 17] (IT)	Italian Society of Paediatrics	English	2018
Japan [18, 19] (JP)	Japanese Society of Paediatric Cardiology and Cardiac Surgery	English	2021
	Japan Pediatric Society	English	2020
Spain [20] (ES)	Spanish Association of Paediatrics	Spanish	2018
South Africa (ZA)	National Department of Health	English	2013
Sweden [21] (SE)	Swedish Association for Paediatric Rheumatology	Swedish	2018
USA [22] (US)	American Heart Association	English	2017

^a^Single hub and access point for pediatric rheumatology in Europe

## Results

### Definitions and diagnosis of KD

According to all guidelines, KD diagnosis still relies on the clinical presentation. Five or more days of fever plus at least 4/5 clinical criteria need to be present for a diagnosis of complete KD. The Japanese guideline [19] included fever among the clinical criteria, resulting that fever is not necessarily mandatory for KD diagnosis.

Most countries define incomplete KD as ≥ 5 days of unexplained fever fulfilling fewer than 4 clinical criteria. However, the minimal number of symptoms required for diagnosis is not specified in all guidelines. Some include the patients’ age in their definition, as younger children are likely to present with fewer symptoms. JP includes the presence of CAA or dilatation in their definition of incomplete KD*.*

In addition to incomplete KD, AR and IT refer to atypical KD in case of “uncommon” symptoms, i.e., meningeal inflammation, seizures, facial paralysis, acute abdomen, pancreatitis, nephritis, cardiogenic shock, cholestatic jaundice, arthritis, and pneumonia.

#### Diagnosis of incomplete KD

None of the guidelines available recommended a specific KD biomarker. The American Heart Association (AHA) developed an algorithm based on expert consensus, for patients with suspected incomplete KD. This algorithm, which combines laboratory findings and imaging for the targeted clinical assessment, is designed to accelerate determining the treatment indication for patients with suspected incomplete KD, but not to differentiate from other febrile diseases. ES, DE, and DK integrated this algorithm in their guidelines [22]. In this context, JP [19] recommends the use of similar laboratory and echocardiographic findings, additionally the hydrops of gallbladder and elevated BNP or NT-pro BNP, but without specific weighting of the values.

#### Definition if incomplete KD

The definition of IVIG resistance varies slightly among different guidelines, with all guidelines considering fever as the main clinical target. Fever should be persistent or recrudescent for at least 24 h according to ZA and 48 h according to SHARE. Fever should resolve within 36 h after having completed IVIG therapy according to the remaining guidelines. Furthermore, laboratory signs of inflammation (SHARE, AR and SE) and clinical signs should resolve significantly (SHARE, SE) to attest to the clear effectiveness of first treatment.

### First-line treatment

#### Intravenous immunoglobulin

All guidelines recommend first-line treatment with IVIG at a dosage of 2 g/kgbw in combination with ASA. IVIG is generally administered over an 8–12-h period. The Japanese guidelines provide detailed information about administration time, depending on the specific IVIG product, emphasizing that according to the Japanese latest nationwide survey it is generally administered 12–24 h [24]. DK recommends a period of 6–8 h. In cases of imminent cardiac failure, IT and SE support a longer period (16–24 h). In addition to these, AR recommends 1 mg/kgbw of diphenhydramine, a first-generation H_1_-antihistamine, 1 h prior to IVIG administration.

#### Acetylsalicylic acid

Dosage of ASA in first-line treatment varies between low (3–5 mg/kgbw/d), moderate (30–50 mg/kgbw/d), and high (80–100 mg/kgbw/d) doses. The US recommends both moderate and high-dose ASA, but no clear preference for either. AR and DK suggest high dose; DE, IT, ES and SHARE recommend a moderate dose. According to all guidelines, ASA should be reduced to low dose (3–5 mg/kg body weight/d) 48 to 72 h after the fever resolved. SE is the only country that proposes starting therapy with low-dose ASA (2–5 mg/kgbw/d), considering a combination with other NSARs or paracetamol. JP explicitly recommends medium-dose ASA in patients presenting with fever and starting with low-dose ASA in patients already presenting afebrile.

#### Corticosteroids (see Table 2)

**Table 2:** Corticosteroids

	**Combination with first-line IVIG** **(High-risk patients only)** ^**a**^	**Combination with 2nd line IVIG (IVIG-resistance)**
Argentina		**IVMP:**30 mg/kgbw/d i.v. over 3 h for 3 days
Denmark		**IVMP:**10–30 mg/kgbw/d i.v. for 3 days *then* **PSL:**2 mg/kgbw/d for 2 weeks
Germany	**PSL:**2 mg/kg/d i.v. then p.o Gradually phased out with normal CRP	Low-risk: **PSL:**2 mg/kg/d i.v. then p.o Gradually phased out with normal CRP High-risk: **IVMP:**10–30 mg/kgbw/d i.v. for 3 days
Italy	**IVMP:**30 mg/kgbw/d i.v. single pulse *or* **PSL**:2 mg/kgbw/d i.v., then p.o Gradually phased out with normal CRP and symptoms	Low-risk: **IVMP:**30 mg/kgbw/d i.v. for 3 days High-risk: **IVMP:**30 mg/kgbw/d i.v. for 3 days *then* **PSL:**2 mg/kg/d i.v. then p.o Gradually phased out with normal CRP/symptoms
Japan	**Recommendation:** **PSL:**2 mg/kgbw/d i.v. in 3 doses; p.o. Gradually phased out with normal CRP/symptoms *or* **Consideration:** **IVMP:** 30 mg/kgbw/d single pulse	**Consideration:** **PSL:**2 mg/kgbw/d i.v Gradually phased out after normalization of CRP *or* **Consideration:** **IVMP:**30 mg/kgbw/d i.v. for 1–3 days
Sweden	**IVMP: **6–30 mg/kgbw/d in 3 doses for 3 days *then* **PSL:**1–2 mg/kgbw/d p.o Gradually phased out (3 weeks)	**IVMP:**6–30 mg/kgbw/d in 3 doses for 3 days *then* **PSL: **1–2 mg/kgbw/d p.o Gradually phased out (3 weeks)
SHARE^b^	1st regime: **IVMP:**0.8 mg/kgbw i.v. for 5–7 days/until normal CRP *then* **PSL:**2 mg/kgbw/d p.o Gradually phases out (2–3 weeks) 2nd regime: **IVMP:**10–30 mg/kgbw (max. 1 g/d) i.v. for 3 days *then* **PSL:**2 mg/kgbw/d p.o. for 7 days/normal CRP, gradually phased our (2–3 weeks)	1st regime: **IVMP:**0.8 mg/kg i.v. for 5–7 days/normal CRP *then* **PSL:**2 mg/kgbw/d p.o., reduced over 2–3 weeks 2nd regime: **IVMP:**10–30 mg/kgbw (max. 1 g/d) i.v. for 3d, *then* **PSL:**2 mg/kgbw/d p.o. for 7 days/normal CRP, gradually phased out (2–3 weeks)
South Africa		**IVMP:**30 mg/kgbw/dose i.v. (duration not specified)
Spain	Severe disease (regimen 1): **IVMP: **30 mg/kg/d i.v. for 3 days *then* **IVMP/PSL:** 2 mg/kg/d i.v./p.o. with normal CRP gradually phased out (2–3 weeks) High risk (regimen 2): **IVMP:**2 mg/kg/d i.v. until defervescence *then* **IVMP/PSL:** as in first regimen	Low risk a)No steroid treatment yet regimen 2 b)With steroid treatment regimen 1 Sever disease/high risk a)No steroid treatment yet regimen 1 b)With steroids further treatment see Table 4
USA	High risk^c^ Corticosteroids^d^ for a course of 2–3 weeks	**IVMP**^e^**: **20–30 mg/kgbw i.v. for 3 days ± *then* **PSL:** 2 mg/kgbw/d; tapered after CRP normalized *or* **PSL:** 2 mg/kgbw/d; tapered after CRP normalized

*IVIG* intravenous immunoglobulin, *IVMP* intravenous methylprednisolone, *PSL* prednisolone, *CRP* C-reactive protein

^a^High risk is either defined as increased risk for IVIG nonresponse and/or development of CAA

^b^SHARE recommends two different regiments without favoritizing one

^c^ “Administration of a longer course of corticosteroids […] may be considered for treatment of high-risk patients with acute KD, when such high risk can be identified in patients before initiation of treatment” [22]

^d^Not specified which kind and dose of corticosteroids should be administered

^e^Alternative to 2^nd^ course of IVIG

Applying corticosteroids in addition to initial IVIG treatment remains controversial. Guidelines with older publishing dates like AR, DK, and ZA do not suggest corticosteroids, and none of the other guidelines recommends general administration in all patients. Most countries recommend first-line corticosteroid therapy only in patients carrying a predictably high risk of being non-responsive to first IVIG treatment (see section on predicting potential IVIG failure and Table 3). Different types and, more importantly, dosages are listed, i.e., high-dose corticosteroid [methylprednisolone (IVMP) as high-dose pulse-therapy (10–30 mg/kgbw/d)] and low-dose corticosteroid [IVMP (0.8–2 mg/kgbw/d) and prednisolone (PSL) (1–2 mg/kgbw/d)]. Low-dose corticosteroids are always administered for a prolonged course entailing a stepwise reduction over several weeks after signs of inflammation have ceased. SHARE and SP optionally propose IVMP pulse before switching to PSL. JP explicitly recommends PSL administration and states that IVMP pulses may be considered as an alternative, which however is not covered by Japanese health insurance for children with KD. (see details in Table 2).

**Table 3:** Specified risk factors in the guidelines

	**Risk factors associated with higher risk of being IVIG-resistant and/or risk of CAA and/or considered for intensified treatment**
Argentina^a^	
Denmark	Not specified
Germany *Intensified therapy*	Recommendation^b^: Initial z-score > 2; ≤ 1 year of age; severe disease (e.g., MAS, shock) Consideration^b^: ≥ 7 years of age; male; pathological laboratory findings (elevated inflammation parameter, elevated liver enzymes, hypalbuminemia, anemia, low sodium); initiation of therapy ≤ 4th or > 14th day of illness
Italy *Intensified therapy*	< 12 months; elevated CRP; elevated aminotransferase level; hypoalbuminemia; severe anemia at disease onset; early development of CAA; signs of MAS or shock
Japan *IVIG resistance/* *Intensified therapy*	Kobayashi score [25], Egami score [26], Sano score [27]
Sweden Intensified therapy	Early anomalies in coronary arteries; < 1 year of age with severe disease^c^; development of/at risk for MAS
SHARE	Kobayashi score ≥ 5 < 12 months; pathological laboratory findings (low serum sodium, elevated liver enzymes, hypoalbuminemia, elevated CRP, low platelet count, falling hemoglobin); features of HLH and shock
South Africa	Not specified
Spain	Severe disease: Shock; < 12 months of age + 2 risk factors for IVIG resistance; CAA or other cardiac pathology High-risk: < 12 months; CRP ≥ 90 mg/l; ESR ≥ 80 mm/h; thrombocytosis ≥ 900 000/mm^3^; hepatic dysfunction; albumin ≤ 2.5 g/dl; sodium ≤ 133 mmol/l; Hb > 2 g/dl under age limit
USA	Not specified

*MAS* macrophage activation syndrome, *IVIG* intravenous immunoglobulin, *CAA* coronary artery aneurysm, *ESR* erythrocyte sedimentation rate, *CRP* C-reactive protein, *Hct* hematocrit, *HLH* hemophagocytic lymphohistiocytosis

^a^Risk factors mentioned but not applied for risk stratification

^b^PSL should be given in recommended risk factors and can be given in considered risk factors

^c^Not specified

#### Prediction of potential IVIG resistance

IVIG resistance raises the risk of developing CAA. In Japan, these children are identified via different risk scores, i.e., Kobayashi [25], Egami [26], or Sano score [27], which its guideline suggests equally. SHARE and the US guideline refer to the Kobayashi score, the US however for Japanese KD children only; none of the other guidelines recommends these scores to select patients for intensified therapy. Most guidelines indicate different individual risk factors including laboratory, clinical, and echocardiographic findings and the patient’s age to indicate intensified primary therapy. AR refers to similar risk factors, however without clear reference to their therapy consequences. For detailed risk factors listed in the guidelines, see Table 3.

#### Cyclosporin A (CsA)

Only the latest Japanese guideline recommends cyclosporin A alternative to corticosteroids for first-line treatment for predicted non-responders.

### Treatment of non-responsive cases

All non-responsive patients should be given a second dose of IVIG at the same dosage. Corticosteroids are the first IVIG additive, second-line treatment of choice, depending on the risk factors specified above (see Table 3) and if corticosteroids have already been applied. IVMP pulse therapy is proposed across all guidelines, at least for those KD patients treated already with corticosteroids. The US guidelines propose IVMP pulse therapy even as an alternative to second-line IVIG. See Table 2 for details on various corticosteroid regimens.

Further therapeutic options include primarily biologicals, mostly proposed as third-line treatment, meaning in KD patients non-responsive to two IVIG and corticosteroid courses. According to the DK, ES, SE, US, and DE guidelines, they may already be considered alternative to a second IVIG therapy. The biologicals proposed include infliximab and etanercept (blocking TNF alpha), and anakinra, an interleukin 1 receptor antagonist. There seem to be no clear arguments supporting any one biological across all guidelines (see Table 4). Canakinumab, an interleukin 1β inhibitor, is only mentioned in the Italian guideline. Ulinastatin (UTI), a urinary trypsin inhibitor, is mentioned by JP and IT. Cyclosporin, methotrexate, and plasmapheresis are mentioned as further treatment options in some guidelines and should only be reserved for patients resistant to the therapy options mentioned above.

**Table 4:** adjunctive therapy

	Anakinra (1–10 mg/kgbw/d)^a^	Infliximab (5–6 mg/kgbw)^a^	Canakinumab (4 mg/kgbw)^a^	Etanercept (0.6 mg/kgbw/week)^a^	Cyclosporin A (Inhibition of calcineurin and increased activity of T cells) (3–5 mg/kgbw/d)^a^	Ulinastatin (human trypsin inhibitor) (15 000–30 000 U/kgbw/d)^a^	Methotrexate (10 mg/m^2^)^a^	Cyclophosphamide (2 mg/kg/d)^a^	Plasma exchange
Argentina		Therapy-resistant		Therapy resistant					
Denmark		2nd line							
Germany	2nd line	2nd line			Mentioned				Mentioned
Italy	Therapy resistant	Therapy-resistant	Therapy-resistant		Drug-resistant KD	Drug-resistant KD; first-line for high-risk patients together with IVIG and ASA	Drug-resistant KD		
Japan					Recommendation: Predicted non-responder, resistance to first dose of IVIG	Consideration: Predicted non-responder	Mentioned		Third-line: contraindication against standard treatment for non-responders; complications like severe infections or KDSS
SHARE		Therapy-resistant							
Spain	2nd line	2nd line			Therapy-resistant			Therapy-resistant	Therapy-resistant
Sweden	2nd line	2nd line							
USA		2nd line			Resistance to second IVIG-infusion, infliximab, or steroids			Therapy-resistant	Therapy-resistant

*KD* Kawasaki disease, *IVIG* intravenous immunoglobulin, *ASA* acetylsalicylic acid, *KDSS* Kawasaki disease shock syndrome

^a^Dosages are represented as the range of dosage recommendation through all guidelines

## Discussion

Following our systematic investigation, we identified KD guidelines from nine countries and one from the SHARE initiative. Five of these guidelines are published in English and are easily available through PubMed. Most countries without national guidelines refer to the US guideline from the American Heart Association [22]. However, the little feedback we received from primarily Asian countries, probably due to language barriers, means that we may have underrepresented some guidelines from Asian countries apart from Japan (see also Fig. 1).

All guidelines still base their KD diagnosis on the classical clinical findings. In terms of incomplete KD, recommendations differ in the number of symptoms and days of illness. The AHA proposes a flow chart adopted by some guidelines to help physicians initiate the treatment of children with suspected incomplete KD. This flow chart however is based on expert recommendations and does not help with differentiating KD from other febrile diseases. Although an enormous investigational effort has been made to improve the KD diagnosis, no specific biomarkers have yet been introduced or even recommended in any of the guidelines we studied.

The evidence on the efficacy of IVIG treatment is well established [5, 6]. All guidelines recommend it be given in a single dosage of 2 g/kgbw. Administration times vary from 8 to 24 h to prevent volume overload leading to cardiac dysfunction.

As in other inflammatory illnesses, high-dose ASA has been used historically and for two main reasons, namely, its anti-inflammatory (moderate- and high-dose) and anti-platelet (low-dose) effects. There is unfortunately no evidence-based ASA effect on either the development of coronary aneurysms [4, 28] or IVIG resistance [29]. In light of this lack of evidence, and given the risk of potential severe adverse reactions such as Reye syndrome, most guidelines now recommend moderate-dose ASA of 30–50 mg/kgbw/d and SE, even starting with the anti-platelet dosage only. Other nonsteroidal anti-inflammatory drugs like ibuprofen widely used in pediatric patients might be an option, but they weaken ASA’s anti-platelet effect [30] –especially important in KD patients suffering severe coronary artery aneurysms. None of the guidelines therefore recommends substituting ASA.

The use of corticosteroids in KD experiences a renaissance. Unlike guidelines with older publishing dates such as AR (2016), DK (2015), and ZA (2013), the more recent ones support administering first-line corticosteroids in “high-risk” cases. Corticosteroids are proposed in two forms: IVMP is given at a dosage up to 30 mg/kgbw/d either as a single pulse or for 3 consecutive days. PSL is usually recommended at a dosage of 2 mg/kgbw/d for a longer time-course and is gradually tapered after normalized CRP and symptoms. Some guidelines recommend starting with IVMP and continuing thereafter with PSL, as illustrated above.

The PSL regime relies on the “RAISE Study” [7], the first randomized controlled trial (RCT) to prove that corticosteroids can be beneficial in both reducing inflammation and in CAA terms. An earlier important RCT from the US [31] investigated a single dose of methylprednisolone in addition to IVIG + ASA detected no significant impact on the CAA outcome. These different outcomes may be explained by the longer corticosteroid treatment, considered to better relieve vascular inflammation, and the RAISE study’s inclusion of “high-risk” patients only. High risk is defined in this context as being at risk of failing to respond to initial IVIG therapy, which in turn is known to be associated with a higher risk of developing CAA. Different “risk scores” such as the Kobayashi et al. [25], Egami et al. [26], and Sano et al. [27] scores have revealed valid predictions of IVIG resistance in Japanese KD children. These scores have unfortunately not yet demonstrated a clinically relevant ability to validly predict the response to standard IVIG therapy outside Japan or in non-Japanese patients [32, 33]. Therefore, according to the Japanese guidelines, all scores are equally recommended and outside of Japan; only the SHARE guideline refers to the Kobayashi-score, as does the US guideline for children of Japanese origin only. The other guidelines compensate for the aforementioned problem by proposing various single factors, each repeatedly demonstrably associated with IVIG-resistance, as to guide intensified therapy such as corticosteroids (see Table 3). None of the available KD guidelines recommends first-line corticosteroid treatment in all KD patients. A currently recruiting multicenter trial across Europe, the “Kawasaki Disease Coronary Artery Aneurysm Prevention trial, (KD-CAAP)” is ongoing and will hopefully help fill the gap of evidence for Caucasian KD children.

Corticosteroids as second-line treatment in case of IVIG-resistance have unfortunately not been proven either to have a significant effect on lowering the CAA rate [34, 35]. Nevertheless, without other evidence-based treatment options, all guidelines propose corticosteroids in this clinical setting despite diverging in dosages and administration times.

Further therapeutic options for children with IVIG-resistant KD are biologicals that focus on blocking the interleukin 1 [36, 37] or TNFα signaling pathway. Data from prospective randomized trials on the TNFα blockers infliximab and etanercept added to first-line IVIG treatment indicated a significant reduced duration of fever and systemic inflammation, but no effect on IVIG resistance [38, 39]. The very recent published KIDCARE trial, investigated infliximab vs. IVIG in IVIG-resistant patients. The infliximab-treated group had a significant shorter duration of fever and hospital stay, reduced need for additional therapy, and less severe anemia; however, infliximab seemed neither to have a better impact on inflammation nor on coronary outcome [8]. Since IVIG-related side effects, i.e., hemolytic anemia, have not been reported in the infliximab-treated group, it may play a more dominant role in future and updated KD guidelines. Further aspects are the reduction of medical costs which may have implications especially for developing countries; i.e., in regard of medication costs, IFX versus IVIG treatment could reduce these to 1/3 of the costs.

Two multi-centric trials are investigating anakinra–the ANAKID [9] (anakinra rescue therapy for patients with existing CAA) and the KAWAKINRA trial (anakinra in IVIG-refractory KD patients). Preliminary data from both trials indicate that the therapy is safe and effective, enabling a rapid reduction in clinical symptoms and inflammation [10, 40]. Although there is no reliable evidence on anakinra yet, some guidelines propose it to be used equivalently to TNFα blocking medications (see Table 4).

CsA inhibiting the Ca2 + /NFAT pathway might contribute to KD susceptibility and CAA development [41]. According to the KAICA trial from Japan, which added CsA to first-line treatment, CAA developed significantly less in the CsA group [11]. Only “high-risk” patients were enrolled in their study. The so for not proven existence of reliable risk identification for non-Japanese children may restrict these results to enter updated KD guidelines even outside Japan. On the other side, Japan being able to rely on evidence regarding effective KD treatment options such as corticosteroids and CsA, the aforementioned biologicals are not part of Japanese treatment recommendations.

The world is currently experiencing a pandemic caused by severe acute respiratory syndrome coronavirus 2 infection (SARS-CoV-2). Although this infection in children is rarely associated with severe disease, in some cases it may lead to a serious inflammatory condition, clinically overlapping with KD, termed pediatric inflammatory multisystem syndrome (PIMS) or multisystem inflammatory syndrome in children (MIS-C). On account of this illness presenting only since recently, the recommendations already provided reflect the currently available evidence of low quality, based on a limited number of case series, retrospective cohort studies, and expert opinions, mostly on basis of KD therapeutic strategies [42, 43]. The variability within the existing international KD recommendation therefor might predispose to international heterogeneous recommendations which need to be considered.

## Conclusion

The clear evidence regarding the first-line IVIG treatment in Kawasaki disease is reflected by consistent treatment recommendations. The less conclusive reliable evidence on additional therapeutic options results in more heterogeneous therapeutic recommendations, which vary also according to the ethnic background and year of publication. High-quality studies are necessary to hopefully increase evidence with time, giving a more solid platform for future recommendation.

## Abbreviations

AHA: American Heart Association
AR: Argentina
ASA: Acetylsalicylic acid
CAA: Coronary artery aneurysm
CRP: C-reactive protein
CsA: Cyclosporin A
DE: Germany
DK: Denmark
ES: Spain
ESR: Erythrocyte sedimentation rate
Hct: Hematocrit
HLH: Hemophagocytic lymphohistiocytosis
IT: Italy
IVIG: Intravenous immunoglobulin
IVMP: Intravenous methylprednisolone
JP: Japan
KD: Kawasaki disease
KDSS: Kawasaki disease shock syndrome
MAS: Macrophage activation syndrome
MCLS: Mucocutaneous lymph node syndrome
MIS-C: Multisystem inflammatory syndrome in Children
PIMS: Pediatric inflammatory multisystem syndrome
PSL: Prednisolone
RCT: Randomized controlled trial
SARS-oV-2: Severe acute respiratory syndrome coronavirus 2
SE: Sweden
US: United States of America
UTI: Ulinastatin
ZA: South Africa
